# Supplementary material for: Experiences of New Zealand Haemodialysis Patients in Relation to Food and Nutrition Management: A Qualitative Study
Source: Nutrients. 2021 Jul 3;13(7):2299. doi: 10.3390/nu13072299 (PMC8308339; doi:10.3390/nu13072299)
Supplement: Supplementary file 1 [file nutrients-13-02299-s001.zip › nutrients-1273405-supplementary.pdf]

Supplementary Material : Experience of New Zealand haemodialysis patients in relation to food and nutrition management: a qualitative study

Rachael McLean [1*], Zhengxiu Xie [1], Vicky Nelson[2], Vili Nosa[3], Hla Thein[4], Audrey Po'e-Tofaeono[3], Rob Walker[5] and Emma Wyeth [2]

Table S1 Interview Protocol

Experience of haemodialysis: Can you tell me a bit about being on dialysis and how you came to be on dialysis.

- How long have you been on haemodialysis?
- Can you tell me how you manage your haemodialysis?
- Do you have dialysis at home or in the clinic?
- Can you tell me a bit about your health in general?
- Do you have other medical conditions? (Diabetes, heart disease, high blood pressure)

Experience of managing nutrition

- Can you tell me about your experiences of organising food drink and meals since you have been on dialysis?
- Can you tell me what you have been told about what how to manage your diet while on dialysis?
- Have you had enough information?
- Was this information understandable / culturally appropriate?
- Where/ who (else) have you had information about diet from?
- How easy is it to stick to this advice?
- What or who has helped? (family/whānau, health services, community groups, WINZ assistance etc)
- What things make it particularly difficult? (Finances, mobility, family pressures)
- What support have you found helpful from healthcare providers?
- What would you need to make it easier to stick to your recommended diet?
- Do you still enjoy your food/ Kai? Does it still taste good?
- What sort of food do you particularly enjoy/ dislike?

How do you think being on dialysis impacts your whānau/ family with respect to eating and providing food?

How does your diet impact on your ability to:

- Socialise?
- Work?
- Participate in cultural events at marae church other…

What advice would you give to someone starting dialysis about managing food/ diet?

Future planning

What other support or help from organisations/agencies would you like to see created for patients and whānau?

Any other comments: Is there anything else you would like us to know?
